# Links between color conspicuousness, territorial behavior, and steroids in a color polymorphic lizard

**DOI:** 10.1093/beheco/arag105

**Published:** 2026-08-26

**Authors:** Cristina Romero-Diaz, Jake A Pruett, Emília P Martins

**Affiliations:** Department of Evolutionary Biology, Museo Nacional de Ciencias Naturales (MNCN-CSIC), José Gutiérrez Abascal 2, Madrid 28006, Spain; Department of Biological Sciences, Southeastern Oklahoma State University, 425 W University Boulevard, Durant, OK 74701, United States; School of Life Sciences, Arizona State University, 427 E Tyler Mall, Tempe, AZ 85287, United States

**Keywords:** animal communication, phenotypic integration, playbacks, signaling, reptile

## Abstract

Discrete color polymorphisms often correlate with other traits, forming suites of characteristics that may be signaled and inform mate-choice decisions and/or dominance interactions with conspecifics. Unraveling the adaptive function and maintenance of stable color polymorphisms thus requires understanding these links and the context in which color traits are used. Here, we conducted a field experiment with robotic models to study the relationship between color morph, territorial behavior,, and circulating hormones, namely testosterone and corticosterone, in a male–male competition framework using the color polymorphic lizard *Sceloporus consobrinus*. We found that yellow and orange male color morphs are conspicuous and can be reliably distinguished by conspecifics, making color differences suitable for morph-specific signaling. Orange males exhibited higher baseline rates of push-up displays and bouts of movement compared to yellow males. Lizards of both morphs strongly increased aggressive display behavior in response to intruder robotic models of either the same or different color morph. However, color morphs did not differ in body size (SVL), body condition or plasma hormonal content (testosterone and corticosterone), suggesting that color could convey information regarding alternative territorial behavior but does not signal differences in individual male “quality” or physiological hormonal state. Color may thus not be sufficient, or the most informative signal in the context of agonistic male contests. Aggressive motion displays may be more effective as a signal of a morph's resource holding potential than static color. The lack of convergence in correlated phenotypes across similarly polymorphic taxa further suggests that patterns of trait integration are rather species-specific.

## Teaser

In male color polymorphic prairie lizards, orange and yellow throats are distinct and conspicuous, yet aggressive motion displays may be more effective at signaling morph differences in resource holding potential/fighting ability than throat color alone. The lack of tight connections between color, individual quality, and hormonal levels makes territorial behavior a more informative signal in the context of agonistic male contests. Considering other similarly polymorphic taxa, the patterns of integration among morphology, physiology and behavior appear to be rather species-specific.

## Introduction

Color polymorphisms, the existence of two or more distinct genetically determined color forms within a single population (Huxley 1955), are widespread in nature (McLean and Stuart-Fox 2014; Wellenreuther et al. 2014) and can be important evolutionary drivers of speciation and diversification (Hugall and Stuart-Fox 2012; Heuer et al. 2024). The main hypotheses explaining the evolution and maintenance of color polymorphisms hinge on discrete visual morphs differing in features other than color, such as morphology, behavior, physiology, or life history, and some form of balancing selection maintaining alternative strategies that achieve similar success (McKinnon and Pierotti 2010). Selection may occur on the morph color, on traits correlated with the color morph, or on combinations of traits that include the color morph (Gray and McKinnon 2007). Thus, to understand the selective forces driving the maintenance of color polymorphisms, it is an essential first step to determine the correlates of color morphs and the social or ecological contexts in which they may function (Roulin 2004). Here, we conducted a field experiment using robotic models with the color-polymorphic prairie lizard (*Sceloporus consobrinus*) to determine the signaling potential of color morph and to study the relationship between color morph, territorial behavior, and circulating hormones, namely testosterone and corticosterone, in a male–male competition framework.

Many animals rely on conspicuous communication such as bright colors, elaborated displays, or loud calls for reproduction (Andersson 1994). Often, the expression of these signals is influenced by an individual's physical state or condition. Thus, these signals may provide relevant information about the quality of the bearer that influences mate-choice decisions or dominance interactions with conspecifics (or both) (Johnstone 1995; Searcy and Nowicki 2005; Számadó 2011; Weaver et al. 2017). In color-polymorphic species, distinct color forms can signal alternative strategies that lead to mating biases (Kokko et al. 2003). In other words, color morph can be a sexually selected signaling trait. For example, female three-spine sticklebacks (*Gasterosteus aculeatus*) rely on male color for mate choice, preferring red-throated to black-throated males (Milinski and Bakker 1990; McKinnon 1995). Red males are typically larger (Tinghitella et al. 2015), are in better condition, and have lower parasitization (Milinski and Bakker 1990) but suffer higher predation (Johnson and Candolin 2017). In the side-blotched lizard, *Uta stansburiana*, male throat color morphs (either blue, orange or yellow) advertise alternative male-competitive strategies (Sinervo and Lively 1996; Corl et al. 2026). Males with orange throats are strongly territorial, more aggressive and dominant to males with blue throats, which are less aggressive and defend smaller territories that overlap with fewer females. Males with yellow throats are nonterritorial; they resemble females and beat orange males by obtaining secretive copulations within their territories. This sneaker strategy, however, is ineffective against mate-guarding blue males. In this system, color conveys information on fighting ability or resource-holding potential to male rivals (Sinervo et al. 2000, 2006).

Alternative behavioral strategies and aggression can be underlain by differences in hormonal profiles between color morphs (McKinnon and Pierotti 2010). For example, testosterone, the primary male sex hormone, is known to modulate aggressive behavior in vertebrates (Book et al. 2001; Wingfield 2005) and plays an important role in male intra-sexual competition and territoriality (Wingfield et al. 1987; Moore 1988). Testosterone also regulates the expression of many sexually selected color traits (eg Casagrande et al. 2011). In side-blotched lizards, the higher aggression, activity and endurance of orange males is linked to higher levels of plasma testosterone compared to yellow and blue males (Sinervo et al. 2000). Corticosterone, a main glucocorticoid secreted primarily from the adrenal cortex in response to various environmental stressors, can also affect color expression (eg Roulin et al. 2008; San-Jose and Fitze 2013). Morphs might differ in their baseline level of corticosterone, like yellow males of the polymorphic lizard *Podarcis melisellensis*, which have lower corticosterone levels than more aggressive orange males (Huyghe et al. 2009), or they may exhibit different hormonal responses to competitive environments, like the more aggressive red-headed male Gouldian finches (*Erythrura gouldiae*), which experience considerably higher increases in corticosterone levels compared to black-headed males in a socially challenging situation (Pryke et al. 2007). In this way, hormonal content may provide a physiological link between color morph and aggressive or territorial behavior.

In this study, we investigated whether throat color morph in male prairie lizards (*Sceloporus consobrinus*) could signal morph differences in individual quality/condition, hormonal levels, and response behavior in a male–male competition context. *S. consobrinus* is one of several species in the speciose genus *Sceloporus* to exhibit a discrete visual color polymorphism (eg Calisi and Hews 2007; Stephenson 2010; Bastiaans et al. 2013; Jiménez-Arcos 2013; Bustos Zagal et al. 2014; Pruett 2017). Lizards of both sexes have either orange or yellow coloration on the throat, chin, and face. A rare third “white” morph lacking either color occurs in some populations (Rand 1990). With the aid of robotic lizard models, we simulated territorial intrusions by yellow and orange morph conspecifics. We quantified male response behavior and tested the relationship between aggression, plasma hormone levels, and coloration. Using a different robotic model for each color morph allowed us to test whether aggressive behavior was conditional on the opponent's morph, as has been reported in other studies with polymorphic species. For instance, in dimorphic three-spine stickleback populations, black-throated males responded more aggressively to red-throated compared to black-throated males (Tinghitella et al. 2015). In male tawny dragon lizards (*Ctenophorus decresii*), a system with four alternative color morphs (orange, yellow, gray, and orange-yellow), yellow males showed higher aggression toward yellow and orange male models than toward a gray model (Yewers et al. 2017). Here, we were interested in answering three specific questions: First, are orange and yellow throats conspicuous in their environment and are morphs distinguishable to a lizard observer? Second, do orange and yellow morphs exhibit and receive different aggressive display behavior? And third, do morphs differ in circulating plasma steroid levels, specifically testosterone and corticosterone? By collecting all three types of data from the same individuals, we ultimately sought to determine whether there is a relationship between color morph, territorial behavior, and hormone profiles in polymorphic *S. consobrinus*. If throat color can function as an intraspecific signal, we expected yellow and orange throats to be conspicuous in their habitats and distinguishable by conspecifics (Guilford and Dawkins 1991; Endler 1992). If these colors carried useful information about morph differences in condition, aggressiveness, or physiological stress in the context of male–male competition, color morphs should correlate to behavior and/or circulating plasma hormones and different morphs may exhibit different response behavior toward yellow and orange competitors.

## Materials and methods

### Study species

**Figure 1:**
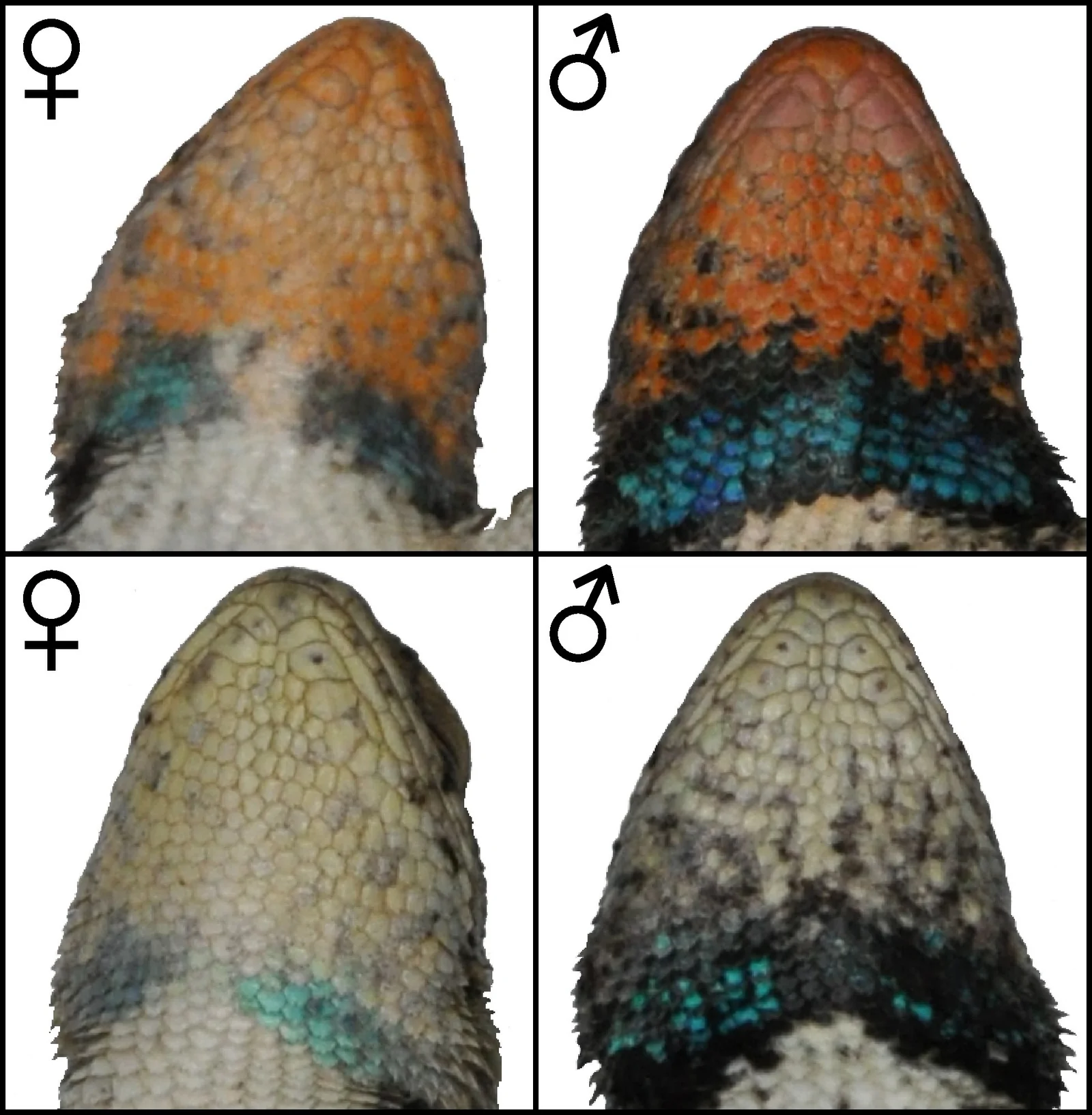
Orange (above) and yellow (below) color morphs of adult female (left) and adult male (right) *Sceloporus consobrinus* from our study population in El Paso County, CO (United States). Photos: C. Romero-Diaz.

The prairie lizard, *S. consobrinus* (syn. *S. undulatus erythrocheilus*), is a small, rock-dwelling lizard with sexual dimorphism (adult male snout-to-vent length [SVL] average: 62 mm, range: 54 to 71 mm; female average: 67 mm, range: 59 to 75 mm) that occupies most of the Southwest Central region of the United States (Leaché and Reeder 2002; Leaché 2009). In our study population, males and females exhibit two alternative morphs that vary in the color of the throat, chin, and face, being either orange or yellow (Fig. 1; Rand 1990). Orange and yellow coloration result from the contribution of three types of chromatophores and the unique presence of an orange-red pteridine pigment (drosopterin) in orange and yellow pteridines (xanthopterin and sepiapterin) in yellow tissues, respectively (Morrison et al. 1995). Color intensity is controlled by activational effects of testosterone and reaches its peak during the breeding season (Rand 1990). However, color morph itself is not under hormone control, nor is it age- or diet-dependent, and it is instead hypothesized to have a genetic basis (Rand 1990, 1992). Male prairie lizards defend territories and competitively exclude other males by engaging in stereotyped push-up displays that they use to signal aggression. During these displays, lizards expose ventrolateral blue patches as well as the polymorphic gular patch (Rand 1991). Lower intensity push-up displays are also performed in the absence of conspecifics to broadcast identity, typically near territorial or home range borders. Chin coloration has been associated with the outcome of agonistic interactions between males. During field observations, Rand (1988, 1992) noted that approximately 90% of aggressive encounters ended with the orange male chasing the yellow male, irrespective of body size. However, this hypothesis was not formally tested, and we lack evidence for the relationship between color morph, aggressive behavior, and naturally occurring levels of steroid hormones.

### Baseline behavior and color quantification

From May to June 2019, we recorded undisturbed individual behavior (“baseline” behavior) in a population situated between the eastern plains and the Colorado Front Range, south of Colorado Springs, El Paso County (CO, United States). We used Canon Vixia HF R800 HD camcorders mounted on a tripod to record from a distance that ranged between 2 and 8 m, depending on habitat conditions. Upon detection, one observer carefully approached a lizard and set up the camera in a place with a direct line of view, subsequently crouched behind the tripod, out of the lizard's view, and started recording up to 15 min of baseline behavior. Baseline recordings thus captured the behavior of focal lizards when they were by themselves, excluding all types of social interactions. Shorter recordings resulted from the lizard moving out of view where we could not follow, hiding in a refuge (eg fleeing from a predator), or the approach of conspecifics that interfered with the behavior of the focal subject. We recorded a total of 49 baseline videos (*n* = 49 males; yellow = 34, orange = 13) ranging from 1.4 to 18.8 min (median: 12.6; average: 11.1). Immediately after, we attempted to capture every lizard using a loop attached to a fishing rod and succeeded in capturing 33 of the focal males (yellow = 24, orange = 9). The lizards were then placed in breathable cloth bags and brought back to the laboratory where we measured SVL (±1 mm) with a ruler, weight (±0.1 g) with a Pesola^®^ scale and took color measurements. In the field, lizard morph was classified by eye by observers (100% agreement between different observers) and later confirmed based on spectral reflectance data (see below). Any lizard that presented an ambiguous morph, ie a yellow throat but orange lips and face, was excluded from the study. Yellow and orange males did not differ in SVL (ANOVA: *F*_1,31_ = 0.02, *P* = 0.896) nor body condition (ie the residuals from the regression of body mass on SVL; *F*_1,31_ = 1.61, *P* = 0.213).

We measured spectral reflectance between 300 and 700 nm of the gular and ventral patches and dorsal skin coloration relative to a white Spectralon reflectance standard (WS-1- SL), using a USB2000 spectrometer (Ocean Optics Inc., Dunedin, FL), a xenon lamp (Ocean Optics PX-2) and a reflectance probe (Ocean Optics R400 UV/VIS) fitted with a black tip cut at a 45° angle. We used Spectra Suite software with the following specifications: scans to average 5, boxcar width 5, and integration time 100 ms. We took three measurements from the middle of the left blue belly patch, three measurements from the center of the gular patch, and three measurements from a centered point of the mid-dorsal surface. We also took three measurements of the spectral reflectance of each of five substrate types on which we found lizards perching (four types of rocks and a log). Measurements from the same substrate type, color patch, and individual were highly correlated (*r* > 0.99 on average) and were averaged for further analyses. Although color measurements were taken between 4 and 5 h after capture, pterin-based orange and yellow lizard coloration is stable over short time periods (Romero-Diaz et al. 2025) and thus not expected to change.

We imported spectral reflectance data into R (v. 4.1.0; R Core Team 2021), using the package pavo (v. 2.7.0; Maia et al. 2019), smoothed spectra with a span of 0.2, and zeroed negative values. The visual system of diurnal lizards (nongekkotan) is tetrachromatic, including four types of single cone photoreceptors: UV-, short-, medium-, and long-wavelength-sensitive photoreceptors (UVS, SWS, MWS, and LWS, respectively) and one class of double cones, with peak sensitivity at approximately the same wavelength as the LWS cone (Fleishman and Font 2019; Osorio 2019). The sensitivity peaks of the different cone classes in *Sceloporus* lizards are unknown; however, spectral sensitivities are remarkably similar across lizard taxa (Fleishman and Font 2019; Osorio 2019). Thus, we investigated throat color dichromatism and color conspicuousness in *S. consobrinus* from the visual system perspective of a lizard using a tetrachromatic receptor-noise limited visual model (Vorobyev and Osorio 1998) and the retinal cone sensitivities of the closely related lizard *Crotaphytus dickersoniane* (Macedonia et al. 2009; Romero-Diaz et al. 2019). We used the pavo functions *vismodel* and *colspace* to extract color metrics for saturation (*r*), hue (theta, *θ*), and luminance (L) (Stoddard and Prum 2008), which have been shown to reliably quantify color differences in similarly shaped spectral curves (Romero-Diaz et al. 2022). Using the *bootcoldist* function, we estimated pairwise chromatic (ΔS) and achromatic distances (ΔL) along with confidence intervals between color morphs and background habitat. We assumed a CIE D65 (standard daylight) illuminant, a Weber fraction of 0.05 and an even cone class ratio (Macedonia et al. 2009). Although thresholds are model and context-dependent, chromatic and achromatic distances can be interpreted approximately in Just-Noticeable-Differences (JND), where 1 JND is predicted to be near the threshold of discrimination, values below 1 JND indicate that two colors are indistinguishable, and values above 1 JND indicate how distinguishable they are, with higher values (eg ≥ 3) indicating increasingly improved discrimination.

We used nontoxic Elmer's Painters^®^ to paint-mark lizards individually with unique combinations of number and color on their dorsal surface, at the base of their tail, and released marked lizards within 24 h at their exact coordinates of capture, which we recorded with a handheld GPS (Garmin^®^ eTrex 30×).

### Visual “playbacks”

**Figure 2:**
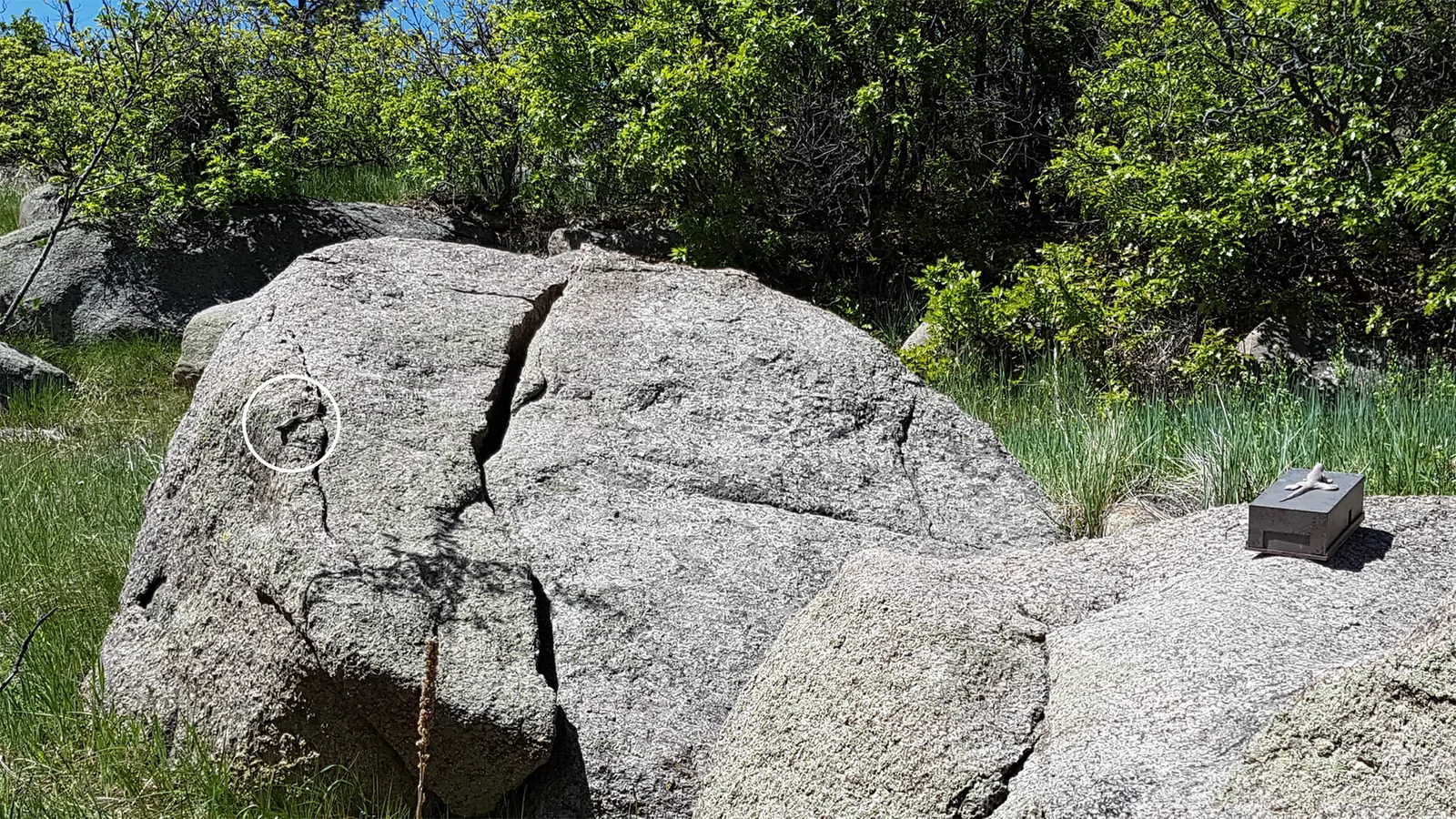
Field presentation of a robotic lizard model (far right) to an adult male *S. consobrinus* (circled, left). Also visible, representative habitat occupied by this species near Colorado Springs (CO, United States): a transitional zone between mixed-grass prairie and mesic oak shrubland, spotted with exposed rocks and fractured boulders on low to moderate slopes facing SE. Photo: C. Romero-Diaz.

We conducted field playbacks (Fig. 2) on marked males after a minimum of 24 h had passed since their release, using two robotic silicon rubber lizard models. Models were cast using a 3D-printed mold of a real *Sceloporus* lizard, scaled to match the size of a medium-sized male *S. consobrinus* (SVL = 52 mm) and mounted on top of a rectangular box (22 × 11 × 7 cm) made of 3 mm-thick acrylic walls, painted like a rock, which contained all electrical and mechanical components. Models were hand-painted to match the dorsal and ventral colorations of a male *S. consobrinus* in two alternative versions: one with orange, another one with yellow throat, chin, and face coloration. Paint color was chosen by eye using *S. consobrinus* photographs (Rand 1991) and the help of a color card reference and validated a posteriori by comparing spectral reflectance data between the model and the lizards (Figure S1). Previous studies show that lizards respond to robotic models in the same fashion that they would respond to a live conspecific (Martins et al. 2005; Smith and Martins 2006; Thompson et al. 2008; Erudaitius et al. 2025). When a marked male was encountered, we would place the robot on a nearby (range: 0.5 to 5 m) surface with a clear direct view and at a similar height from the ground to that of the male (making the model's left side face him), turn the robot on and retreat to a covert position. After 60 s, the robot would start moving, reproducing the push-up display characteristic of *S. undulatus* (Carpenter 1978) in a loop, pausing for 5 s in between subsequent displays. While no data on the push-up/headbob display pattern of *S. consobrinus* is available, *S. undulatus* and *S. consobrinus* were considered a single polytypic species (“*S. undulatus*”) until recent molecular analyses determined that they represented different lineages (Leaché and Reeder 2002). Thus, we expect their push-up displays to differ only slightly, as is the case among populations of the same species (Martins et al. 1998; Romero-Diaz et al. 2023).

We recorded the subject lizard's behavior continuously for 5 to 6 min after the beginning of a trial, which was defined by the moment the robot started displaying. We presented each lizard with both robotic models within a timeframe ranging from 1 to 6 d, randomizing the order of presentation for a total of 46 trials with 23 unique subjects (2 playbacks per subject). A few marked lizards for which we had baseline recordings were not encountered again or could only be presented with one of the models and thus were excluded from the behavioral analyses.

### Hormone assays

Once visual playbacks were completed, we measured circulating levels of plasma testosterone (T) and corticosterone (CORT) from male *S. consobrinus* in the field (*n* = 23; yellow = 16, orange = 7). We collected blood samples from the orbital sinus of re-captured lizards, at least 24 h after the end of their second playback, with heparinized capillary tubes (Microcaps, Drummond Scientific, Broomall, PA). Bleed time, measured as the time passed from the moment of capture until the blood draw was complete, ranged from 1.8 to 6 min (mean: 3.6 min; median: 3.8 min). Previous studies indicate that collecting blood samples within 10 min following disturbance is adequate to obtain baseline measurements of plasma CORT concentration in the congener *S. undulatus* (Tylan et al. 2020). Likewise, capture and handling stress up to 180 min do not alter circulating androgen levels in *S. virgatus* (Hews and Abell Baniki 2013). Thus, we presume that plasma T and CORT levels reported here are representative of baseline levels in free-ranging male *S. consobrinus* during the mating season. Blood samples were centrifuged after collection, and the resultant plasma was diluted in 100% ethanol (1:10 dilution of plasma to ethanol) and stored at −20 °C until analysis. We assayed hormone levels with commercially available enzyme-linked immunosorbent assay (ELISA) kits (testosterone ELISA kit [catalog # ADI-900-065] and corticosterone ELISA kit [catalog # ADI-900-097], Enzo Life Sciences, Farmingdale, NY) following protocols from Wada et al. (2007).

We first optimized the kits for our study species by analyzing serial dilutions of plasma pooled from 12 males from our study population. Pooled plasma was evaporated under a flow of nitrogen, resuspended in assay buffer, and then stripped of steroid hormones with a charcoal solution. Aliquots of the stripped sample were then spiked with known concentrations of testosterone or corticosterone standards and 2 concentrations (1.0 or 2.0%) of a steroid displacement reagent. The resultant dilutions were assayed in duplicate using separate plates for each hormone. Consistent with our work on other *Sceloporus*, the optimal dilution was 1:80 lizard plasma and assay buffer with 2.0% concentration of steroid displacement reagent for either hormone.

We assayed samples for individual males by combining 4 µl of plasma with 4 µl steroid displacement reagent diluted in 312 µl of assay buffer. Samples were pipetted in duplicate (100 µl aliquots) onto 96 well plates, and we followed the assay procedures provided by the kit manufacturer for each hormone. Once the assays were complete, we added a stop solution to each well and plates were immediately read on a SpectraMax^®^ Plus 384 microplate reader (Molecular Devices, Sunnyvale, CA). Sample concentrations were determined with a 4-parameter logistic curve-fitting program (Softmax Pro 5.2TM). We calculated individual lizard plasma testosterone and corticosterone values as the average of their respective duplicate measures. A single plate was used to measure plasma testosterone for all samples, and the intraassay coefficient of variation was 13.2%. This coefficient of variation was measured as the average across all samples in the plate, which were run in duplicate. The testosterone antibody has moderate cross-reactivity with 19-hydroxytestosterone (14.6%) and androstendione (7.2%) and low cross-reactivity (<1.0%) with dehydroepiandrosterone and estradiol. The two tested steroids with highest cross-reactivity, 19-hydroxytestosterone and androstendione, may have neural effects via aromatization (Callard et al. 1977; Hahn et al. 1985; Wade 1997). For plasma corticosterone, samples were assayed on 2 plates with intra-assay coefficients of variations of 7.5% and 16.5% and an inter-assay coefficient of variation of 11.9% (calculated from replicate standards across the plates). The corticosterone antibody has moderate (21.3%) cross-reactivity with deoxycorticosterone, and low (<0.5%) cross-reactivity with progesterone, testosterone, tetrahydrocorticosterone, aldosterone, cortisol, pregnenolone, and β-estradiol. We are unaware of any study of plasma levels of deoxycorticosterone in *Sceloporus*, the one steroid with substantial cross-reactivity to the corticosterone antibody of this kit. Deoxycorticosterone, a mineralcorticoid, can be neural or adrenal in origin and can be converted to neuroactive metabolites in acute stress (Reddy 2006); hence, further work is needed to attribute any behavioral changes that we document directly to plasma corticosterone elevations.

### Statistical analyses

From videos, the same person (C.R-D) scored counts of (i) chemosensory behavior, namely, the summed number of tongue flicks, substrate licks, and gular pumps; (ii) display behavior (push-ups, push-up displays, headbobs, and full shows), and (iii) bouts of locomotion. See Table S1 for a detailed description of each behavior. Scoring was done blind with respect to robot morph, but focal lizard morph was visible on video and thus recognizable. We compared rates of baseline and response behavior between orange and yellow morphs (*n* = 23; yellow = 14, orange = 9) using the counts of chemical acts, push-ups, push-up displays, and bouts of locomotion as response variables and the log of trial duration (in s) as the offset term with negative binomial generalized linear mixed models in R (function glmer.nb) (Bates et al. 2015). The use of an offset term allowed us to measure rates adjusting for variation in trial duration. In the models, we included the factors “trial type” (baseline/playback) and “morph” (orange/yellow), their interaction, body size (“SVL”) as a covariate, and “lizard ID” as a random effect. Headbobs were too infrequent to analyze, and full shows only occurred during playback trials. We determined the significance of model predictors using Type II Wald chi-square tests (function Anova). We ran post-hoc contrasts on significant interactions with the function testInteractions, package phia (De Rosario-Martínez et al. 2015), and adjusted *P*-values with the Holm–Bonferroni method. We then analyzed the data for both playback types (orange and yellow robotic model) separately from baselines, so that we could test morph differences in response behavior toward either version of the robotic model (“robot” [orange/yellow]), which were not present in baseline trials, and whether the distance between the focal male and the robot (“F–R distance”) would influence behavior. In this model, we were also able to include the interaction between morph and robot. We confirmed the lack of overdispersion in the data for all negative binomial GLMMs.

We tested for differences in plasma testosterone and corticosterone levels between morphs using linear models (function lm) that included “morph” as a factor, body size (“SVL”) as a covariate and their interaction term. We also tested the correlation between plasma hormones and color metrics (luminance, saturation and hue), and rates of baseline behavior in all males for which we had data for both (*n* = 23; yellow =16, orange = 7). We examined model residuals to confirm that they conformed to the homoscedasticity and normality assumptions of linear regression with Bartlett's tests and quantile–quantile plots.

## Results

### Orange and yellow morphs are conspicuous and can be discriminated by conspecifics

**Figure 3:**
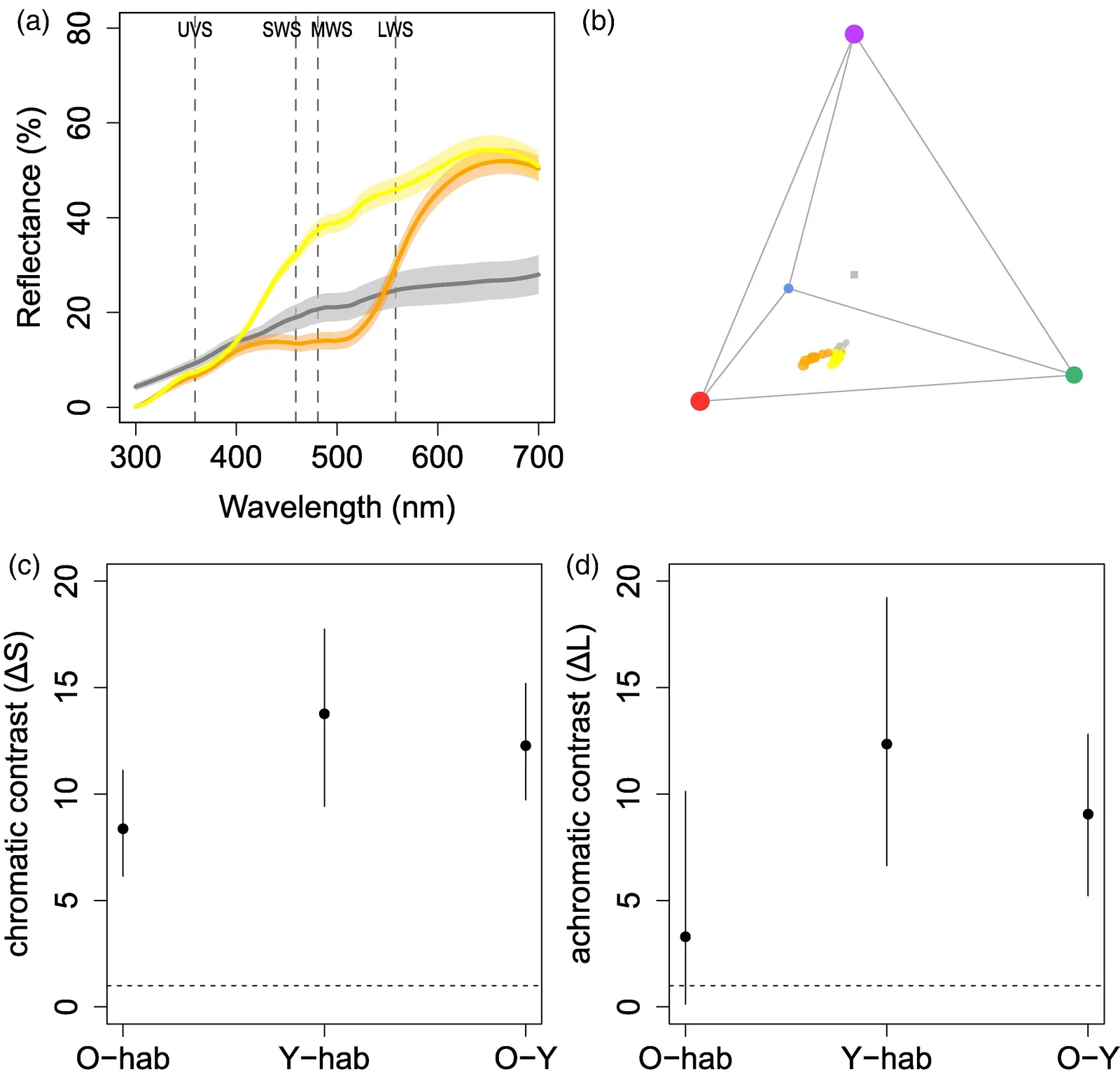
Top row: Mean spectral reflectance of male throat color morphs in *S. consobrinus*: yellow (“Y”, yellow line/dots), orange (“O”, orange line/dots), as well as background habitat (“hab”, grey line/dots), along with their positions in tetrahedral color space based on a model of lizard vision. Vertical dashed lines indicate peak receptor sensitivities of *Crotaphytus dickersonae* (UVS: 359 nm; SWS: 459 nm; MWS: 481 nm; LWS: 558 nm; (Macedonia et al. 2009). Bottom row: bootstrapped, noise-corrected chromatic and achromatic distances (in units of just-noticeable differences, JNDs) between color morphs and habitat background.

We found that male throat coloration from *S. consobrinus*’ orange and yellow morphs were similarly and highly conspicuous in their habitats (Fig. 3c and d), showing average color distances larger than 8 JNDs. The exception was the low achromatic contrast of orange throats, which made them likely indistinguishable from the background habitat in achromatic terms under bad viewing conditions (eg low light). Orange and yellow throats also differed significantly in all three color descriptors (Table S2; yellow males had higher luminance, less saturation, and larger theta [less red]) and were clearly distinguishable from one another to a lizard observer (Fig. 3b–d). In contrast, ventral blue and dorsal coloration were likely indistinguishable between different morphs (Figure S2; ventral CC: 1.9 JNDs [95% CI: 0.5, 10.5], ventral AC: 1.4 JNDs [95% CI: 0.1, 7.2]; dorsal CC: 1.7 JNDs [95% CI: 0.4 to 3.5], dorsal AC: 3.7 JNDs [95% CI: 0.3, 8.2]). Ventral and dorsal patches were less than, or as reflective as the background habitat.

### Lizards from different color morphs exhibit different baseline but similar response behavior

**Figure 4:**
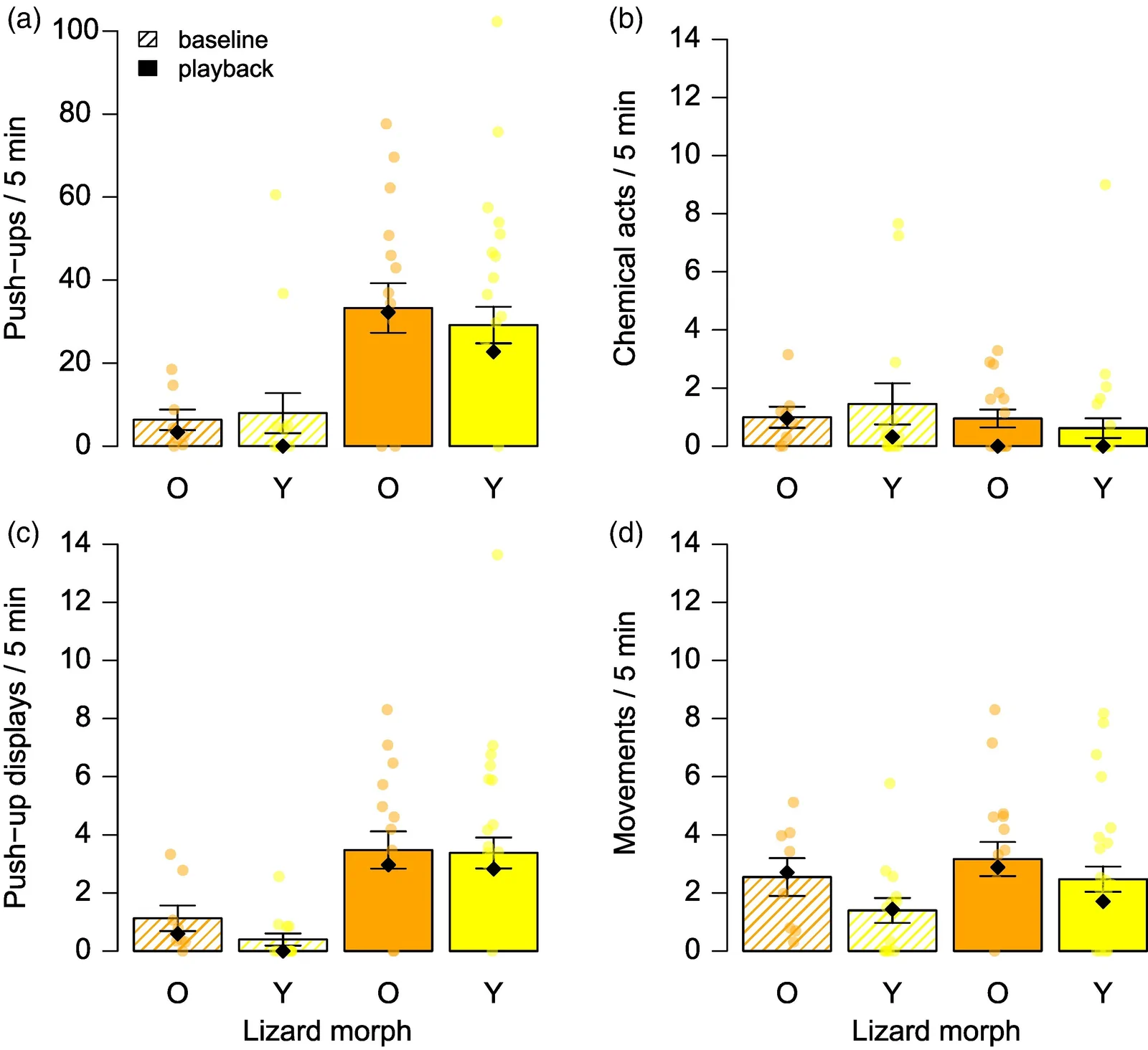
Differences between undisturbed behavior (“baseline”) and in response to robotic lizard models (“playback”) of male orange (O) and yellow (Y) morphs of *S. consobrinus*. Shown are mean rates per 5 min ± SE, medians (black diamonds) and individual data points (circles, with horizontal jitter for clarity).

Males from both color morphs increased aggressive display behavior in response to lizard models. They more than tripled the rate of push-ups and push-up displays performed (Table 1; Fig. 4a and c). A significant cross-over interaction between morph and trial type showed that orange males performed more push-up displays than yellow males in baseline trials (Post-hoc: *χ*^21^ = 5.56, *P* = 0.037), resulting in a steeper increase in push-up display rate for yellow males in playback trials (Fig. 4c; O: *χ*^2^_1_ = 12.74, *P* < 0.001; Y: *χ*^2^_1_ = 35.75, *P* < 0.001). In contrast, we found no differences in the rate of chemical acts between trial types nor between orange and yellow morphs (Table 1; Fig. 4b). Lizards from both morphs displayed higher rates of movement (bouts of locomotion) in playback than in baseline trials, and orange lizards moved more than yellow lizards overall (Table 1; Fig. 4d). Body size had no influence on any lizard behavior (Table 1).

**Table 1:** Results from negative binomial generalized linear mixed models on male *S. consobrinus* behavior.

Response	Trial type	Morph	SVL	Trial type × morph
	*χ* ^2^_1_ (*P*)	*χ* ^2^_1_ (*P*)	*χ* ^2^_1_ (*P*)	*χ* ^2^_1_ (*P*)
Push-ups	**25.89 (<0.001)**	0.40 (0.527)	0.54 (0.460)	0.03 (0.858)
Push-up displays	**43.19 (<0.001)**	0.70 (0.402)	0.02 (0.892)	**5.11 (0.024)**
Chemical acts	3.12 (0.077)	0.16 (0685)	0.98 (0.322)	1.10 (0.294)
Movements	**4.13 (0.042)**	**4.08 (0.043)**	0.16 (0.692)	1.33 (0.248)

Significant values in bold.

When we analyzed response behavior in playback trials separately, we found that different color morphs had similar rates of chemosensory, display behavior and bouts of locomotion in the presence of either orange or yellow robotic models (Table 2). The only exception was that males from either morph performed more movements in the presence of yellow robotic models, compared to orange models (estimate ± S.E. = 0.37 ± 0.19; Table 2). The distance between the focal subject and the robotic model had no effect on lizard behavior during playback trials (Table 2).

**Table 2:** Results from negative binomial generalized linear mixed models on male *S. consobrinus* behavior during playbacks.

Response	Morph	Robot	SVL	F–R distance	Morph × robot
	*χ* ^2^_1_ (*P*)	*χ* ^2^_1_ (*P*)	*χ* ^2^_1_ (*P*)	*χ* ^2^_1_ (*P*)	*χ* ^2^_1_ (*P*)
Push-ups	0.45 (0.501)	0.47 (0.494)	0.19 (0.658)	0.23 (0.634)	0.26 (0.607)
Push-up displays	0.04 (0.848)	1.69 (0.194)	0.49 (0.485)	0.01 (0.939)	0.55 (0.457)
Chemical acts	0.98 (0.322)	0.09 (0.769)	2.23 (0.135)	0.00 (0.961)	2.19 (0.139)
Movements	1.49 (0.221)	**4.11 (0.043)**	1.48 (0.223)	0.40 (0.525)	0.10 (0.747)

Significant values in bold.

### Color morphs are not underlain by different levels of plasma hormones

**Figure 5:**
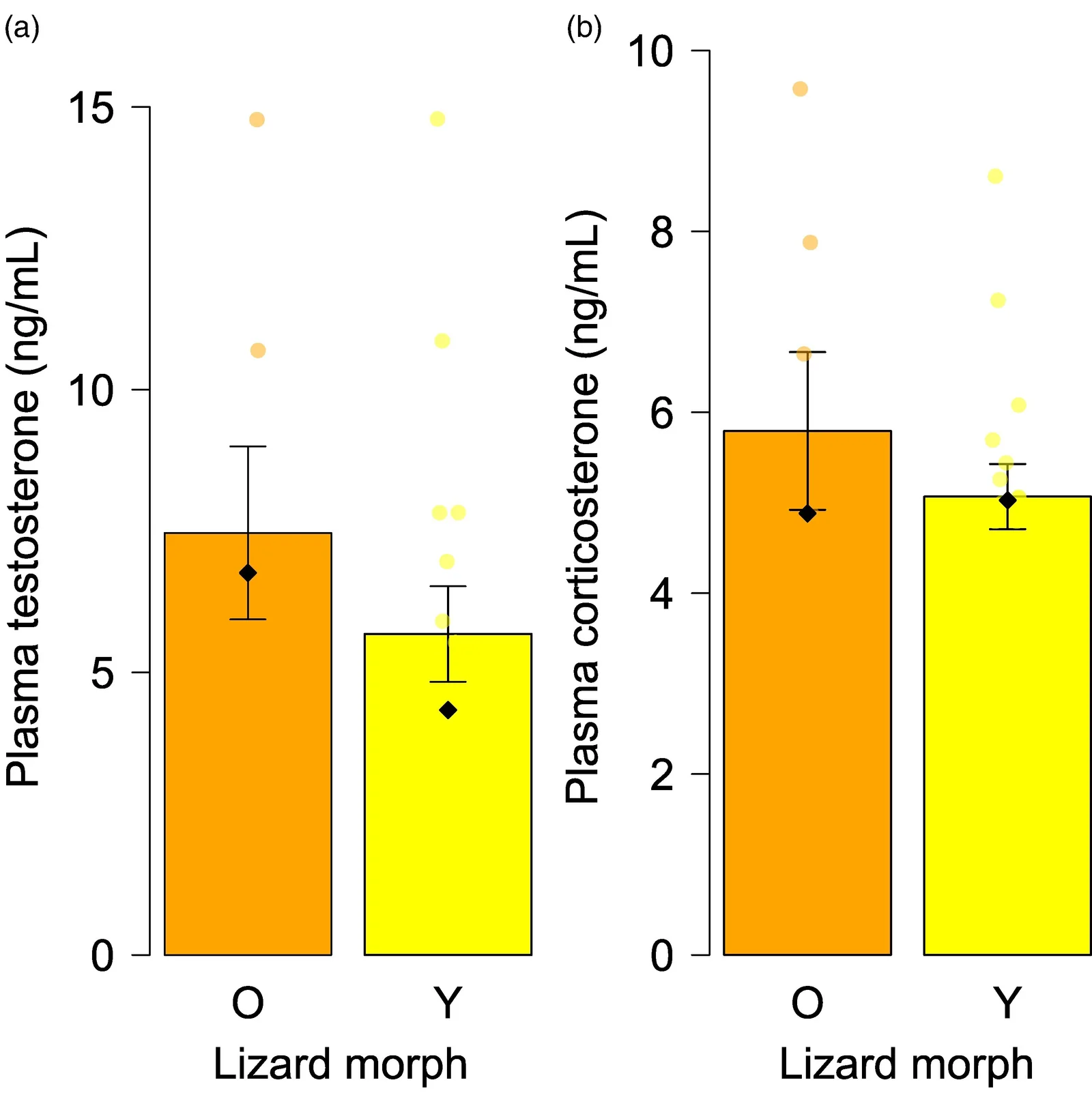
Circulating plasma testosterone and corticosterone did not significantly differ between male *S. consobrinus* from different color morphs (O = orange, Y = yellow). Shown are means ± SE, medians (black diamonds) and individual data points (circles, with horizontal jitter for clarity).

We found that circulating T and CORT levels did not differ between color morphs (Fig. 5), nor were predicted by body size (T full model: *F*_3,19_ = 0.54, *P* = 0.661; CORT full model: *F*_3,19_ = 0.52, *P* = 0.672; Table S3). In addition, the color metrics luminance, saturation, and hue were unrelated to plasma T (all *P* > 0.361) or CORT (all *P* > 0.108; Figure S3). We also found no linear relationship between circulating plasma T (all *P* > 0.247) or CORT (all *P* > 0.057) and baseline behavior (Figure S4). However, morphs differed in the sign of the relationship between plasma CORT concentration and bouts of movement, which was positive in yellow males (ie higher-CORT yellow males increased movement) and negative in orange males (ie higher-CORT orange males moved less), leading to a significant morph × CORT interaction (*χ*^2^_1_ = 6.16, *P* = 0.013). Plasma CORT levels were also not correlated to bleeding time (*F*_1,21_ = 0.30, *P* = 0.588).

## Discussion

Discrete color morphs often correlate with other morphological, physiological, behavioral, or life-history traits. In a male–male competition context, male color polymorphism may thus advertise alternative strategies to rivals. Here, we found that orange and yellow *S. consobrinus* throat color morphs are conspicuous in their environments and can be discriminated by conspecifics, making color differences suitable for morph-specific signaling. Morph differences emerged on display behavior, specifically in the form of push-up display rate, and bouts of movement, both of which were higher in orange compared to yellow males. In contrast, color morphs did not differ in body size (SVL), body condition, or plasma hormonal content (testosterone and corticosterone), suggesting that color could convey information regarding alternative territorial behavior but does not signal individual male “quality” or physiological hormonal state.

As expected, lizards responded strongly to the presence of robotic models (Martins et al. 2005; Kelso and Martins 2008; Thompson et al. 2008; Erudaitius et al. 2025), increasing aggressive display behavior (eg push-ups, full-shows), yet chemosensory behavior remained unaffected (Fig. 4; Figure S5). This pattern is in line with previous studies in the *Sceloporus* genus where staged territorial intrusions using a predominantly visual stimulus (a live conspecific) elicited a mainly visual response (Romero-Diaz et al. 2021). When undisturbed, orange lizards performed higher rates of push-up displays and overall higher rates of locomotion bouts than yellow lizards, suggesting differences in territorial behavior between morphs, reminiscent of other taxa (eg lizards: Sinervo and Lively 1996; Mandavilli et al. 2025; birds: Horton et al. 2014; Searcy and Nowicki 2021; fish: Lehtonen 2014). Push-up rate however, was similar between morphs, which may suggest intraspecific variation in the structure of the push-up display, such as in the number of push-ups per display (Crews 1975; Martins 1991; Martins et al. 1998), although this possibility needs further study. In field observations of natural aggressive encounters, Rand (1991) described alternative facultative behavioral strategies for orange and yellow morphs, surmising that orange males could be “territorial” or “nonterritorial,” but yellow males were always nonterritorial. Territoriality was characterized by the defense of smaller home ranges with higher female overlap where male owners would successfully exclude all intruders. Nonterritoriality involved occupying a larger home range with lower female density where the male was not dominant in all aggressive encounters with rivals. Our results support this hypothesis, since a higher push-up display and movement rate in orange males indicate higher patrolling activity and broadcast signaling, key components of territorial marking and defense in *Sceloporus* lizards (Ruby 1978; Martins 1993; Sheldahl and Martins 2000; Romero-Diaz et al. 2023). However, both morphs responded similarly to robotic models during playbacks, suggesting that the degree of territoriality is more likely a consequence of circumstance (eg the outcome of male–male encounters), rather than alternative fixed strategies. In other words, yellow males also exhibit strong territorial defense behavior but might be dominated more frequently by more aggressive and active orange males (Rand 1991). These results are reminiscent of recent findings by Corl et al. (2026), suggesting that alternative competitive strategies in yellow and blue morphs in *U. stansburiana* may be plastic and depend on the availability of territories.

Bouts of locomotion comprised all sorts of short- and long-range uninterrupted movements longer than 1 body length, including approaches and charges against the robot in playback trials, which might also be indicative of higher aggression toward intruders by orange males. However, this distinction between different types of movement was not made explicit within the data and thus not analyzed. Lizards of either morph were more active in the presence of yellow robotic models (Figure S6a), which might suggest that these models were more approached overall, compared to orange models, although they were not the target of more aggressive displays than were orange models (Table 2, Figure S6b). This may also suggest a tendency to gather more information before escalating aggression in the presence of yellow males (Ossip-Drahos et al. 2018). This result is in line with a field experiment that manipulated focal male morph using orange and yellow paint and a mirror to study morph differences in agonistic behavior (Rand 1991). Rand found no differences in display behavior (push-ups), irrespective of the lizard's morph and the color it was painted, but he found that yellow-painted yellow males were more likely to approach or “chase” their mirrored image than orange-painted yellow males. Unlike in other studies (eg Decourcy and Jenssen 1994), the distance between the focal male and the model (intruder) had no influence on aggressive response behavior, and thus behavioral differences toward yellow models cannot be attributed to the perceived proximity of the threat. Higher movement rates by orange males might also reflect higher visitation frequency to females within a male's territory (Rand 1988). Since territoriality in phrynosomatid lizards is mainly maintained with reproductive purposes (Ruby 1978; Stamps 1983; Abell 1997), these behavioral differences might also point to alternative reproductive strategies between orange and yellow males. The absence of a relationship between behavior and body size and lack of differences in SVL and body condition between morphs further indicate that coloration may be a better predictor of dominance (aggressive behavior) and resource holding potential than body size in this species (Rand 1991).

Interestingly, neither color nor behavioral differences between the morphs were underlain by dissimilar hormone profiles. In hormone supplementation experiments with *S. consobrinus*, Rand (1992) demonstrated that the administration of testosterone increased the intensity of throat color expression but did not change their color morph. In agreement with this, here we found no significant differences in baseline plasma T concentration between orange and yellow males (Fig. 5a), indicating that the observed morph differences in color and territorial aggressive behavior were not driven by differences in circulating testosterone. This result contrasts to those found in other polymorphic lizards such as *S. parvus*, where circulating T levels were lower in blue-white compared to blue-yellow throated males (Pruett 2017), or the tawny dragon, where orange males showed higher levels of plasma T than gray and yellow males (Yewers et al. 2017). White- and tan-striped plumage morphs of white-throated sparrows (*Zonotrichia albicollis*) also differed in plasma T, and these differences underlie alternative breeding strategies and different levels of aggression (Hews et al. 2015). Our results resemble those found in polymorphic *Podarcis muralis* lizards, where color morphs showed equivalent mean levels of plasma T across the breeding season (Sacchi et al. 2017) with the caveat that *P. muralis* does not exhibit morph differences in aggressive behavior (Abalos et al. 2016, 2020), and thus plasma T differences are not necessarily expected.

We also found no differences in plasma CORT concentration between color morphs, and although CORT was unrelated to color metrics, we found evidence that it co-varied with the rate of bouts of locomotion in opposite directions for orange and yellow males (Figure S3). This could imply that orange males become more likely to stay put (and defend territories) with increasing physiological stress, whereas yellow males become more likely to run away. CORT is linked to increased antipredator behavior in *Urosaurus ornatus* in different ways among color morphs (Thaker et al. 2009). In *U. stansburiana*, alternative morph phenotypes seem to result from pleiotropic effects of regulatory genes coupling hormonal differences, coloration and behavior (Corl et al. 2026). In *Sceloporus* lizards, seasonal changes in circulating levels of T are tied to increased levels of territorial aggression, which are maximal during the breeding season (Moore 1986; Moore and Marler 1987; Smith and John-Alder 1999). However, in the species that have been studied, plasma T and CORT do not seem to change shortly following aggressive encounters, despite these encounters affecting behavior (Moore 1987; Klukowski and Nelson 1998; but see Smith and John-Alder 1999 for lizards in captivity), suggesting that circulating hormones do not closely track differences in behavior. In polymorphic *S. pyrocephalus*, females with red and grey striped gular regions had higher T concentrations compared to those with yellow and dark gular stripes (Calisi and Hews 2007). Moreover, color expression has sometimes been shown to positively correlate with T and CORT levels, such as throat brightness in polymorphic *S. grammicus* (Argaez et al. 2021). In contrast, blue patch coloration, which did not differ between orange and yellow morphs, seems unrelated to T and CORT in six other *Sceloporus* species (Zúñiga-Vega et al. 2021). The emerging pattern is that of a weak link between circulating hormones and color polymorphic expression in this species.

Strong throat color conspicuousness and morph differences in aggressive behavior suggest that color morph can function as a signal of fighting ability in *S. consobrinus*. However, the overall lack of differences in the behavioral response toward orange and yellow robotic models that were otherwise the same (eg in size, behavior) suggest that perhaps color is not sufficient, or not the first or the primary source of information during agonistic male contests (Erudaitius et al. 2025). In *S. minor*, polymorphic dorsal coloration had no impact in the outcome of male contests. Winners were rather predicted by increased aggressive behavior and larger body size (García-Rosales et al. 2021). In *S. consobrinus*, morphs do not differ in body size, thus aggressive display behavior is likely the most informative predictor of fighting ability (Rand 1991; Abalos et al. 2024). Perhaps motion signals, which more easily attract the attention of receivers and can be perceived at longer distances compared to static signals (Tan and Elgar 2021), are more effective signals in a context of territorial defense. We should note, however, that our sample size, particularly for orange males, was low on account of this morph being less abundant in our study population, which may have affected the power to detect morph differences. Nevertheless, differences in throat coloration were enough to elicit more movementaround yellow models, perhaps because they are somewhat easier to discriminate from the background (Fig. 3).

In conclusion, we found that *S. consobrinus* color morphs are conspicuous and distinguishable but unrelated to size, body condition or circulating steroid hormone levels. Behavioral differences between morphs emerged on display and movement behavior, indicating different territorial strategies, that were however largely not conditional on the morph of the opponent, nor predicted by testosterone or corticosterone levels, nor the distance to the model. Even though throat color is suited to signal dominance and resource holding potential in male aggressive encounters, it may not be used to discriminate opponents of different morph. Based on these and other findings stemming from polymorphic systems that have been studied in some detail, no general pattern of integration between color morph, physiology and behavior across taxa can be described. Instead, correlated phenotypes seem to be rather species-specific, and color morphs that are linked to alternative behavioral strategies yield different behavioral responses in different contexts.

## Supplementary Material

arag105_Supplementary_Data

## Data Availability

Data used in this study is available in figshare: https://doi.org/10.6084/m9.figshare.30870680. Analyses reported in this article can be reproduced using the data provided by Romero-Diaz et al. (2026).
