# Supplementary material for: Links between color conspicuousness, territorial behavior, and steroids in a color polymorphic lizard
Source: Behav Ecol. 2026 Aug 26;37(5):arag105. doi: 10.1093/beheco/arag105 (PMC13540734; doi:10.1093/beheco/arag105)
Supplement: Supplementary file 1 [file arag105_supplementary_data.pdf]

Supplementary material for

Links between color conspicuousness, territorial behavior, and steroids in a color polymorphic lizard

Cristina Romero-Diaz[1*], Jake A. Pruett[2] & Emília P. Martins[3]

[1]Department of Evolutionary Biology, Museo Nacional de Ciencias Naturales (MNCN-CSIC), Madrid 28006, Spain.

[2]Department of Biological Sciences, Southeastern Oklahoma State University, Durant OK 74701, USA

[3]School of Life Sciences, Arizona State University, Tempe AZ 85287, USA

*Corresponding author: cromero@mncn.csic.es

Tables

Table S1: Description of the behaviors analyzed in this study.

Behavior	Description
tongue flick	A rapid protrusion and retraction of the tongue toward the air or a quick opening and closing of the mouth with partial protrusion of the tongue.
substrate lick	A directed tongue-flick where the protruded tongue contacts the substrate.
gular pump	A brief extension of the floor of the buccal cavity and throat with the mouth closed.
push-up	The lifting and lowering of the upper or full body by the extension of the legs (either two or four), oftentimes performed alongside up-and-down movements of the head.
push-up display	A series of push-ups performed in succession.
headbob	The up-and-down movement of the head performed in isolation, without the lifting of the body.
full show	The sustained exposure of the abdominal patch by the dorsolateral flattening of the abdomen, an arched back and extended legs, typically but not necessarily done while performing push-ups.
bout of locomotion	Uninterrupted displacement that involved moving a distance larger than one body length.

Table S2: Morph differences in color descriptors. Statistics from linear models including "morph" (orange / yellow) as a fixed factor and "SVL" as a covariate. SVL was only significant in the analysis of luminance (negatively correlated, not shown). Yellow males had higher luminance, less saturation and larger theta (less red) than orange males. *n* = 23.

Color descriptor	Estimate (yellow)	*F*	d.f.	*P*
luminance (*L*)	2222.4	19.64	1,20	< 0.001
saturation (*r*)	-0.04	11.77	1,21	< 0.001
hue (θ)	0.19	345.2	1,21	< 0.001

Table S3: Morph differences in circulating plasma hormones. Statistics from linear models including "morph" (orange / yellow) as a fixed factor, "SVL" as a covariate and their first order interaction. $n = 23$.

	morph		SVL		morph × SVL	
Hormone	F (P)	d.f.	F (P)	d.f.	F (P)	d.f.
T	1.29 (0.271)	1,19	0.01 (0.914)	1,19	0.33 (0.572)	1,19
CORT	0.70 (0.414)	1,19	0.63 (0.435)	1,19	0.12 (0.727)	1,19

Figures

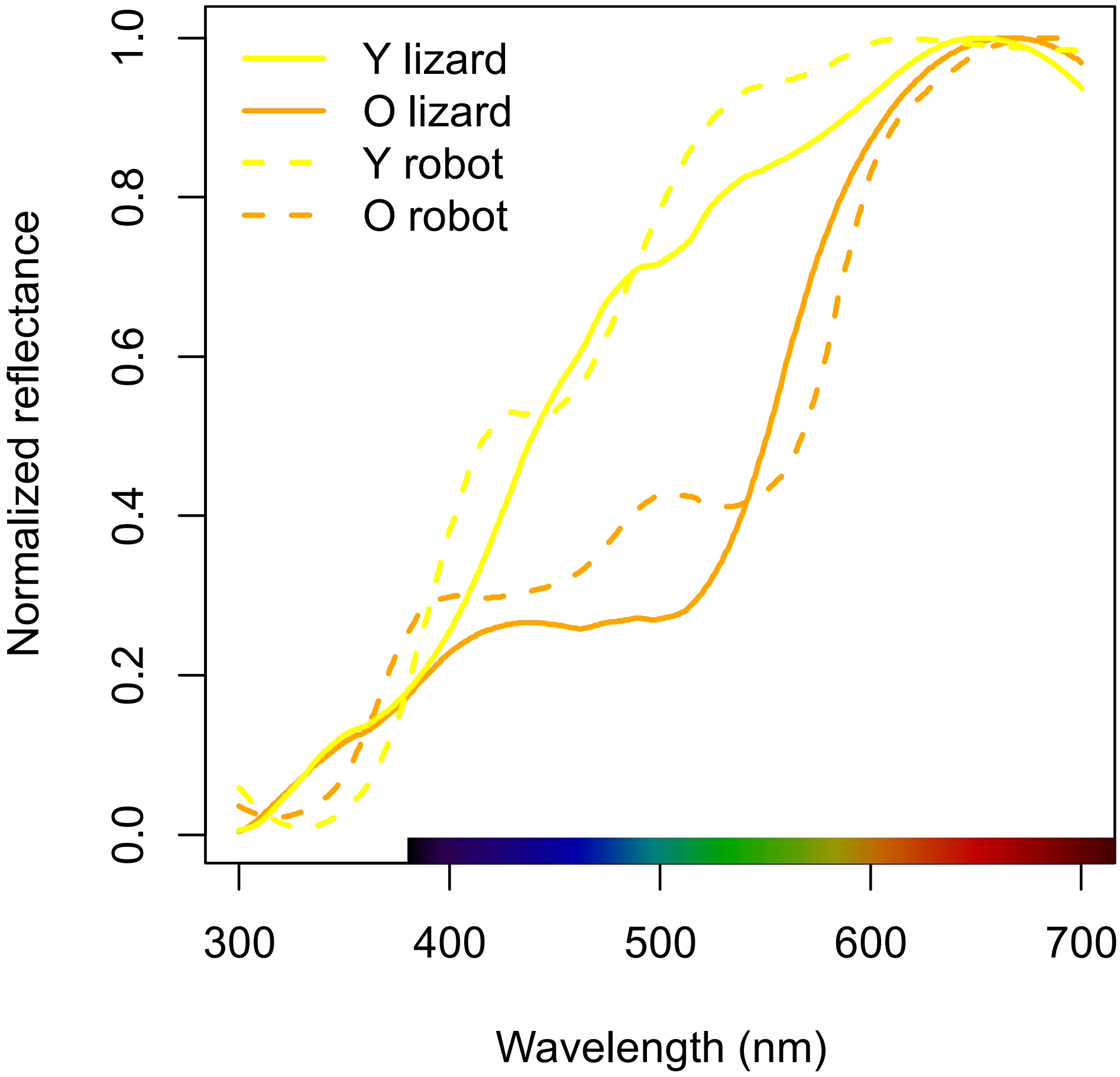

Figure S1: Spectral reflectance of yellow and orange throats from male *Sceloporus consobrinus* (solid lines, see Fig. 3a) and hand-painted robotic models (dashed lines). Lines for lizards represent averages and n = 1 for models.

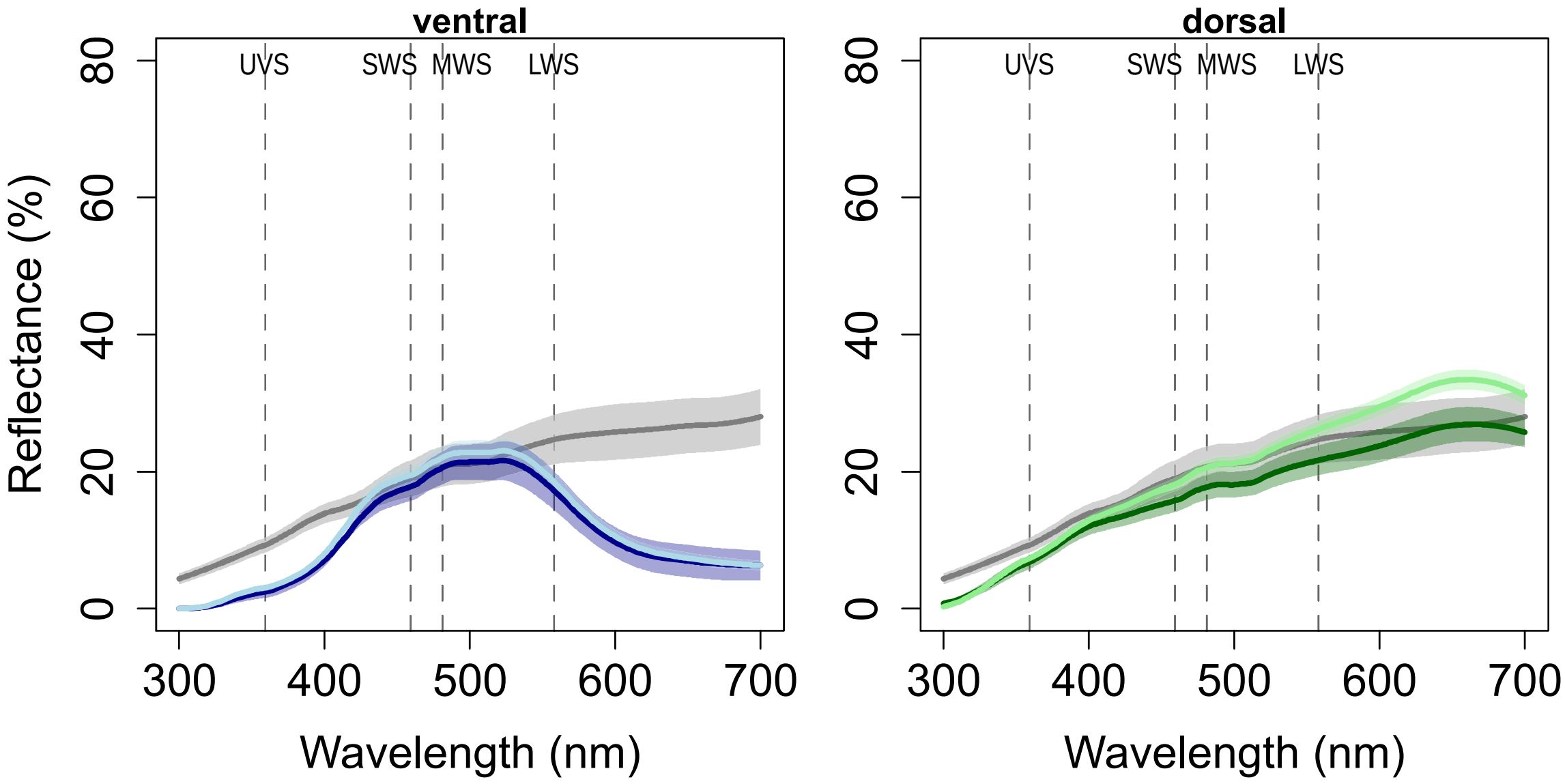

Figure S2: Spectral reflectance of ventral blue and dorsal surfaces from male *S. consobrinus* of different color morph: dark blue and dark green lines depict reflectance for orange morph males (n = 7 and n = 8 for ventral and dorsal spectra, respectively) and light blue and light green depict the average for yellow morph males (n = 21 and 24 for ventral and dorsal spectra, respectively). Grey lines correspond to mean spectral reflectance of the background habitat.

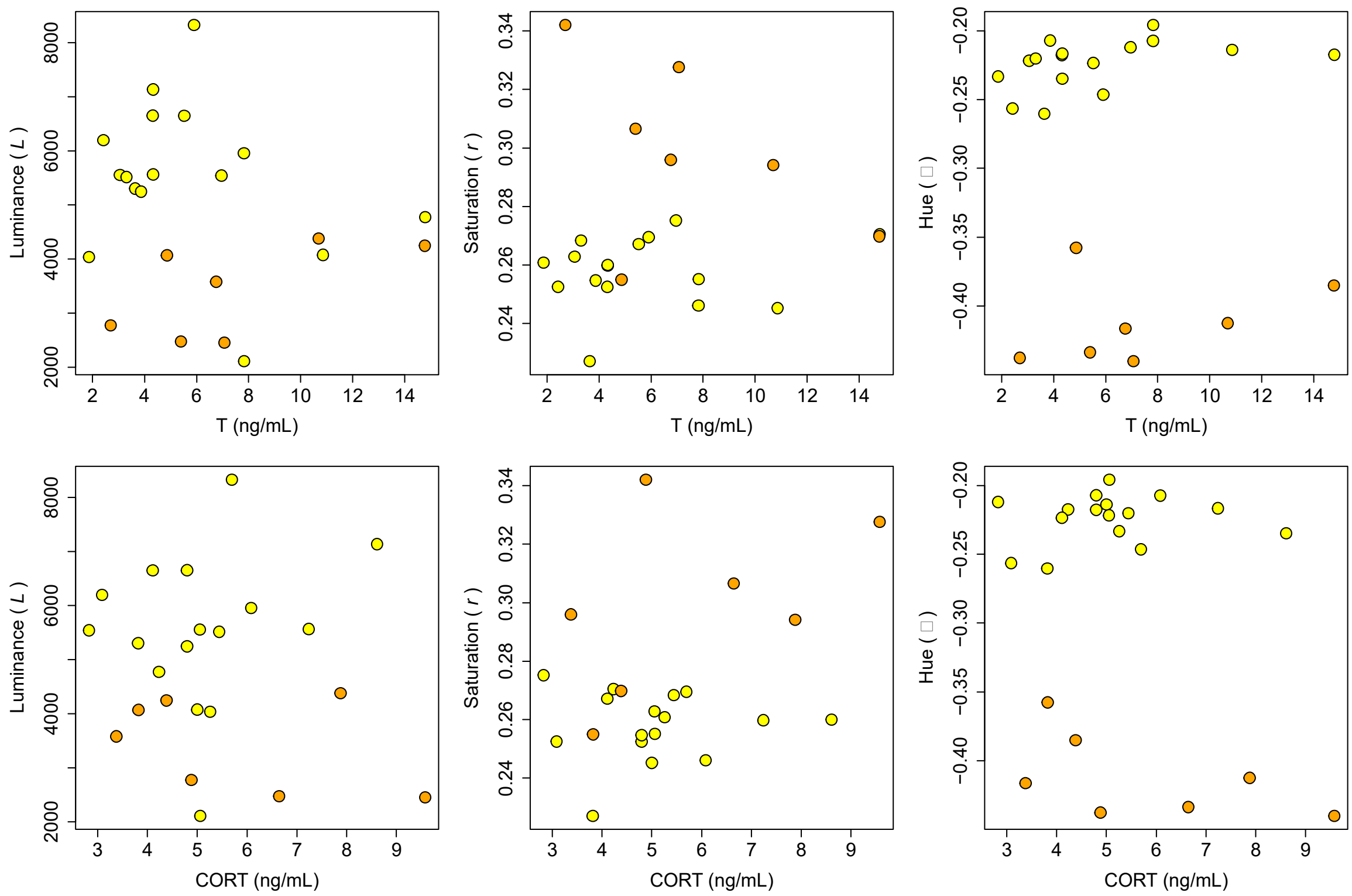

Figure S3: Neither circulating plasma testosterone (T) nor corticosterone (CORT) levels correlated with throat color metrics (luminance, saturation, and hue). Each point corresponds to an individual male lizard and throat morph is indicated by color. $n = 23$.

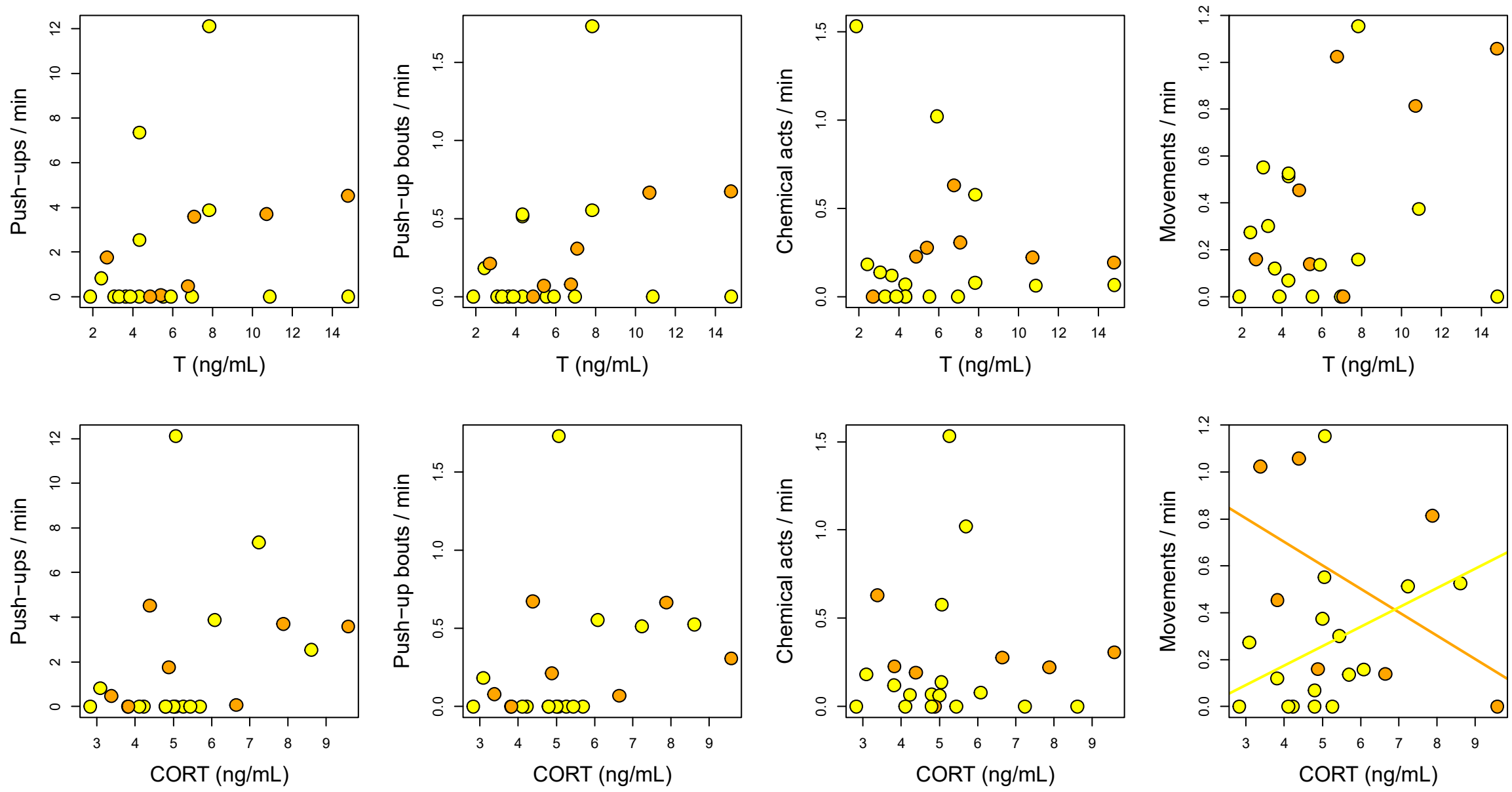

Figure S4: Circulating plasma testosterone (T) and corticosterone (CORT) levels were largely unrelated to baseline behavior in male *S. consobrinus*. Each point corresponds to an individual male lizard and throat morph is indicated by color. $n = 23$.

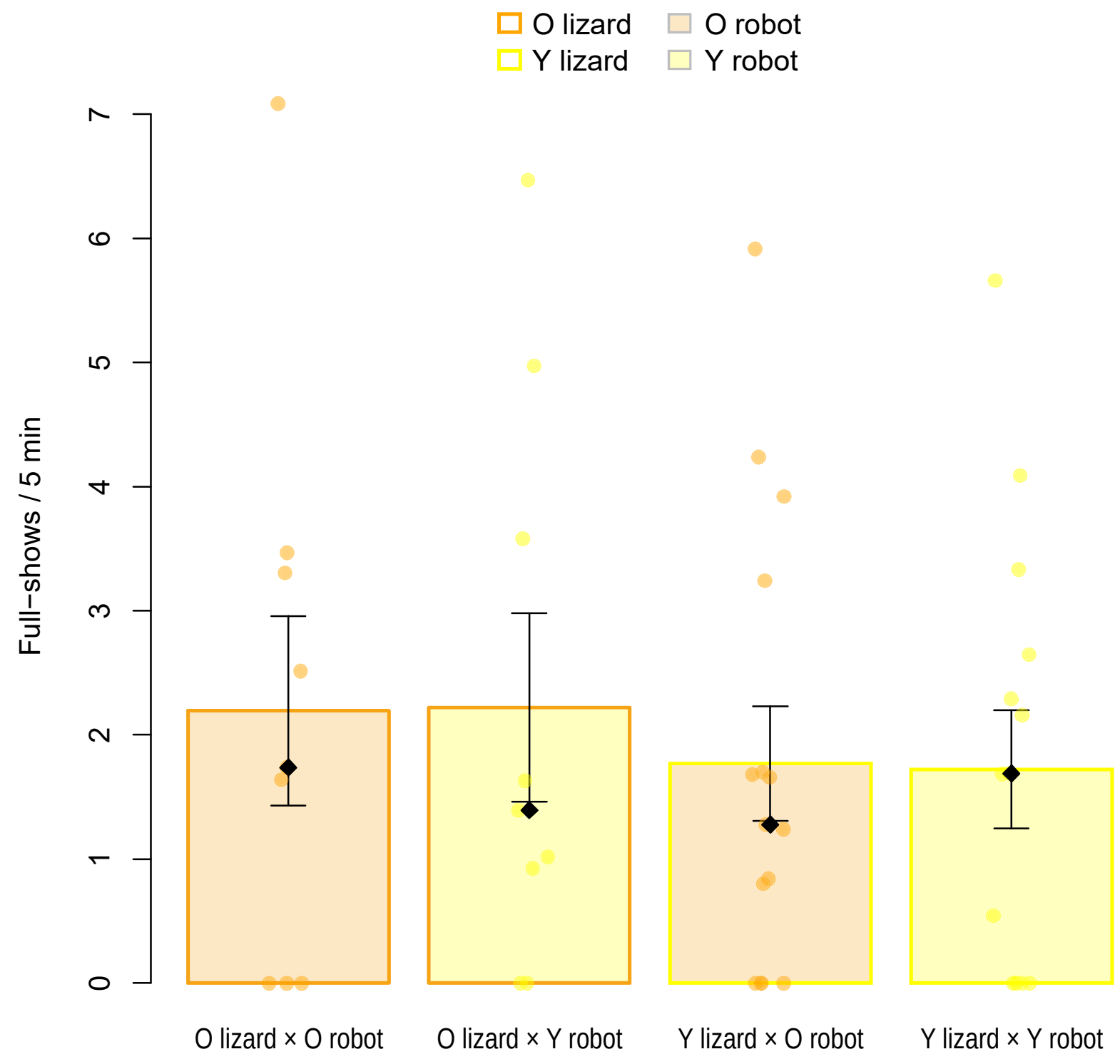

Figure S5: Mean rates per 5 min ± SE of full-show displays performed in response to robotic lizard models by orange (O lizard) and yellow (Y lizard) *S. consobrinus* males toward orange (O robot) and yellow (Y robot) robotic models. Also shown are medians (black diamonds) and individual data points (circles, with horizontal jitter for clarity). No full-shows were observed in baseline trials.

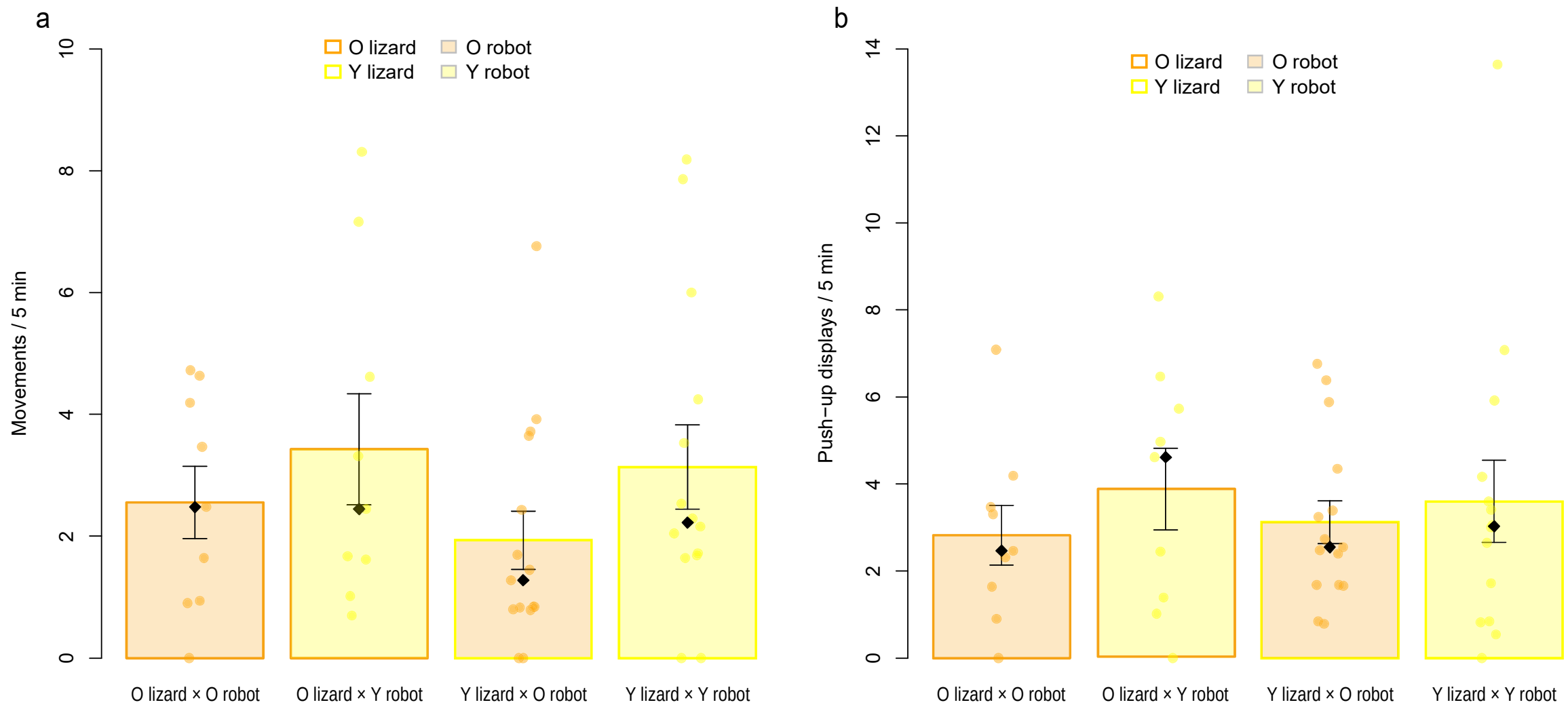

Figure S6: Mean rates per 5 min ± SE of movements (a) and push-up displays (b) performed in response to robotic lizard models by orange (O lizard) and yellow (Y lizard) *S. consobrinus* males toward orange (O robot) and yellow (Y robot) robotic models. Also shown are medians (black diamonds) and individual data points (circles, with horizontal jitter for clarity).
